# Fluorescent Microscopy-Based Detection of Chitin in Intact *Drosophila melanogaster*

**DOI:** 10.3389/fphys.2022.856369

**Published:** 2022-04-26

**Authors:** J. Flaven-Pouchon, B. Moussian

**Affiliations:** ^1^ Interfaculty Institute of Cell Biology, University of Tübingen, Tübingen, Germany; ^2^ Instituto de Neurociencia, Universidad de Valparaíso, Valparaiso, Chile; ^3^ INRAE, CNRS, Institut Sophia Agrobiotech, Université Côte d'Azur, Nice, France

**Keywords:** chitin, FB28, cuticle, drosophila, appendages

## Abstract

Chitin is the major scaffolding component of the insect cuticle. Ultrastructural analyses revealed that chitin adopts a quasi-crystalline structure building sheets of parallel running microfibrils. These sheets called laminae are stacked either helicoidally or with a preferred orientation of the microfibrils. Precise control of chitin synthesis is mandatory to ensure the correct chitin assembly and in turn proper function of cuticular structures. Thus, evaluation of chitin-metabolism deficient phenotypes is a key to our understanding of the function of the proteins and enzymes involved in cuticle architecture and more generally in cuticle biology in insects. Usually, these phenotypes have been assessed using electron microscopy, which is time-consuming and labor intensive. This stresses the need for rapid and straightforward histological methods to visualize chitin at the whole tissue level. Here, we propose a simple method of chitin staining using the common polysaccharide marker Fluorescent brightener 28 (FB28) in whole-mount *Drosophila melanogaster*. To overcome the physical barrier of FB28 penetration into the cuticle, staining is performed at 65°C without affecting intactness. We quantify FB28 fluorescence in three functionally different cuticular structures namely wings, dorsal abdomens and forelegs by fluorescence microscopy. We find that, as expected, cuticle pigmentation may interfere with FB28 staining. Down-regulation of critical genes involved in chitin metabolism, including those coding for chitin synthase or chitinases, show that FB28 fluorescence reflects chitin content in these organs. We think that this simple method could be easily applied to a large variety of intact insects.

## Introduction

The polysaccharide chitin is a major component of the insect cuticle. Chitin fibers are bundled building higher order chitin microfibrils that in turn are arranged in parallel forming horizontal sheets called laminae. Within the cuticle, laminae are stacked either helicoidally or with a preferred direction of the microfibrils along the apical-basal axis of the cuticle. Synthesis and organization of chitin within the cuticle requires the membrane-inserted glycosyltransferase chitin synthase, chitin binding proteins and chitin modifying enzymes including chitinases, chitin deacetylases and the DOMON domain Knickkopf (Knk) proteins (Moussian, 2010; Muthukrishnan et al., 2012). Impairment of chitin synthase activity by introduction of mutations into the respective gene, by reduction of its transcript levels by RNA interference (RNAi) or by administration of insecticides targeted against the chitin synthase are lethal. Likewise, the activity of chitin synthesis, organization and modification associated proteins and enzymes is essential for insects. Hence, chitin organization shapes the architecture of the cuticular tissue and is the support for the large diversity of cuticular proteins (CPRs) characterized by the presence of the conserved chitin binding domain R&R. Nevertheless, this chitin binding domain is not sufficient to fix cuticular proteins and the cuticle undergoes a sclerotization step after its synthesis. This step is induced by the so-called tanning hormone Bursicon and its receptor DLGR2 encoded by the *rickets* (*rk*) gene (Baker and Truman, 2002; Luo et al., 2005). Following its release, oxidized catechols transported to the cuticle leading to the formation of adducts between cuticular proteins and chitin resulting in a stable chitin/CPRs network (Schaefer et al., 1987; Andersen, 2010). Evaluation of chitin synthesis, organization and modification deficient phenotypes is central to the understanding of the function of the proteins and enzymes involved in chitin metabolism and more generally the cuticle biology. Usually, these phenotypes are analyzed by transmission electron microscopy. As this method is rather labor intensive and time consuming, a rapid and simple protocol would allow fast and efficient scoring of the phenotypes. Since decades, chitin staining has been carried out in various Fungi and Arthropods using Calcofluor M2R, also known as Fluorescent brightener 28 (FB28) (Harrington and Hageage, 2003). Brighteners were originally used in textile industry and FB28 interest as a stable fungal-cell staining agent was firstly demonstrated by M. Darken (Darken, 1961; Darken, 1962). FB28 was then shown to bind longitudinally to polysaccharide microfibers including chitin (Harrington and Raper, 1968; Herth and Schnepf, 1980). In Insects, chitin staining using FB28 has been limited to thin organs sections such as epidermis, wings or gut (Pesch et al., 2016; Dong et al., 2020; Zhang et al., 2021) or embryos (Moussian et al., 2005). On the other hand, various chitin quantification methods have been applied to insects albeit limited to whole body extracts analysis (Farnesi et al., 2012; Chen et al., 2013; Henriques et al., 2020). Here, we propose a simple, unexpansive and rapid method to stain with FB28 the insect model *Drosophila melanogaster* without prior dissection, thereby allowing chitin detection and quantification at the whole tissue level.

## Methods

Visualizing chitin content at the level of a whole tissue is of interest since cuticular structures can be particularly complex and variable in an insect especially in the imago. The simple method we propose here was developed to address this need. FB28 has been routinely used in a large panel of organisms to visualize chitin extracellular matrices. Novel chitin staining reagents, namely Direct Yellow 98 and Direct Red 23, have been proposed as more stable and specific alternatives than FB28 but their current price limits their use for large-scale applications (Hoch et al., 2005; Sviben et al., 2020). Thus, we used FB28 as a chitin marker for whole-body staining. Unfortunately, FB28 does not penetrate intact *Drosophila melanogaster* at 25°C (Supplementary Figure S1). We had previously been successful in staining intact *Drosophila* using Eosin Y by varying the staining temperature (Wang et al., 2016). Hot Eosin Y experiments showed that epicuticular lipids are differentially distributed on the insect exoskeleton and that they play a major role to prevent xenobiotics penetration. Following the same rationales, we developed a Hot FB28 staining protocol to facilitate FB28 penetration and therefore chitin detection at the tissue level. Importantly, our method must address four major concerns: 1) FB28 intensity could reflect FB28 penetrability through the epicuticular lipidic barrier. 2) FB 28 intensity could reflect autofluorescence in the blue spectrum (resilin content). 3) FB28 intensity could be affected by cuticular pigmentation. 4) FB28 intensity could reflect the density of cuticular protein covalently bound to chitin. Assessing these four concerns likely improves the faith that FB28 intensity could precisely reflect chitin content in a given cuticular structure. If so, FB28 intensity should be affected by the down-regulation of chitin-metabolism genes. In this study, we provide several clues to address these concerns.

### Hot FB28 Staining of Intact Flies

Groups of 5 48 h-old females were housed in 2 ml plastic vials, kept at −20°C until staining. Flies were washed during 5 min either with chloroform or distilled water. Chloroform was shown to be the most efficient polar solvent to remove cuticular lipids (CHCs) and facilitate dye penetration (Wang et al., 2016). Then, flies were briefly rinsed with 70% ethanol and then distilled water. Once the 2 ml vials were dry, 1 ml of PBST containing FB28 was added and vials were transferred on a warming agitator at 300 rpm during 20 or 60 min using different temperature (50 °C, 60 °C or 65 °C). After staining, flies were bathed 5 times (5 min each), 1 time in PBST and 4 times in distilled water. During these washes, vials were placed in a vertical agitator. Then, wings, dorsal abdomens or forelegs were dissected in PBS and mounted in Polymount medium (Polyscience, Inc.^©^). GAL4 drivers with differential expression in epidermis for these three structures have been extensively characterized and allow a direct comparison in the same individual. For abdomen staining, the abdomen content of 2 h-old flies was removed before staining. After staining, dorsal abdomen was simply detached from the thorax and mounted.

### FB28 Intensity Quantification

Samples were imaged under a Nikon binocular (Nikon AZ100) using Lumencor light engine^®^ illuminator and UV filter (Nikon EX330-380 DM 400, BA 420). The same acquisition parameters were kept for all pictures. The objective used was Nikon AZ plan Fluor 5x1. Pictures were taken using three different expositions for the different tissue (900 ms for wings, 300 ms for dorsal abdomen, 600 ms for forelegs). Pictures were then analyzed with ImageJ. Color channels were split, and the blue channel was used to measure mean grey values of the measured zones. Measures zone were selected according to *engrailed*, *pannier* or *distalless* expression pattern as illustrated in the respective main figures. Polygons selection tool was used to delimit each zone. For wings, polygons strictly followed the *engrailed* expression pattern excluding the anterior veins and proximal veins network thereby focusing on inter vein areas (polygons selection in Figure 2A). For abdomens, measurments were made in the third tergite excluding the melanized posterior part (red rectangle in Figure 3A). Moreover, polygon selections in *pnr +* area were not taken in the very center of the segment to avoid melanization traces and to stay as much as possible in the same plan as the *pnr-*polygonal selection. For legs, polygon selections were taken in tarsal segments, tibia and femur (illustrated in Figure 5A) avoiding articulations enriched in resilin, a component which auto fluoresces in the blue spectrum (Lerch et al., 2020) To evaluate correlation between FB28 intensity and cuticle pigmentation in pigmentation mutants, pigmentation was measured using mean grey value (MGV) in the entire (indicated as a red rectangle in Figure 4A) third tergite as previously described (Flaven-Pouchon et al., 2020). In order to produce a more intuitive score for which darker cuticles had larger values, the final score was obtained by subtracting MGV from 250. Thus, the score was obtained using the following formula:

Pigmentation=−MGV+250

The value of 250 was chosen arbitrarily because it was the smallest value that produced positive values for all readings.

### Statistics

FB28 intensity measurements are shown using scattergrams representing all data of a group and its mean (red cross). Statistically significant differences were determined using one-way ANOVA followed by a Tukey HSD post hoc analysis when samples were normally distributed or by a Kruskal Wallis test followed by Conover-Iman post hoc analysis when strong deviation from normality was detected in each sample (Shapiro test and QQ plot). All statistical analyses were performed using XLSTAT 2016^©^.

## Material

### Fly Husbandry and Crosses

Fly stocks were raised on standard cornmeal/molasses/yeast food and maintained at 22 °C. Unless noted, fly stocks were obtained from the *Drosophila* Bloomington stock center (BL; Bloomington, United States) and the Vienna Drosophila Research Center. Strains used in this study were *w* ^1118^, *en*-GAL4 (BL 30564), *pnr*-GAL4 (BL 3039), *dll*-GAL4 (BL 3038), UAS-*cht6* RNAi (BL 54823), UAS-*cht10* RNAi (BL 57160), UAS-*knk* RNAi (VDRC 106302), UAS-*kkv* RNAi (VDRC 100327). UAS-*tBur* was kindly provided by A. Kopin. As down-regulation of these genes are mostly lethal during embryological and larval development, all GAL4 drivers used in this study were crossed preliminarily with *tub*-GAL80^ts^ to restrict RNAi expression to metamorphosis. All crosses were performed at 25 °C, progeny was raised at 18 °C until pupariation and then placed at 29 °C. The *yellow* ^1^ and *w* ^*1118*^ strains derived from stocks of the Nüsslein-Volhard laboratory; the *tan* ^*3*^ (BL 132) mutant stock was purchased from the Bloomington stock center, the *ebony* mutant strain was made by CRISPR/Cas9 in the background of wild-type Tübingen 2018 flies (Mokeev et al., 2021).

### Reagents

Fluorescent brightener 28 (1 mg/ml) in PBST (Tween 1%) solution kept at 4°C in dark place. Purchased from Sigma Aldrich.

Chloroform chemical grade >98%. Purchased from Sigma Aldrich.

Distilled water.

Polymount medium polyscience Inc.^©^.

### Equipments

Eppendorf Thermomixer content (300 RPM) for FB28 staining.

Vertical rotator to rinse the samples.

Dissection material: Dumont forceps Cat5, Vanna’s spring scissors (Fine science tool^©^).

## Results

### FB28 Applied at High Temperature Leads to Homogenous Wing Staining

**FIGURE 1:**
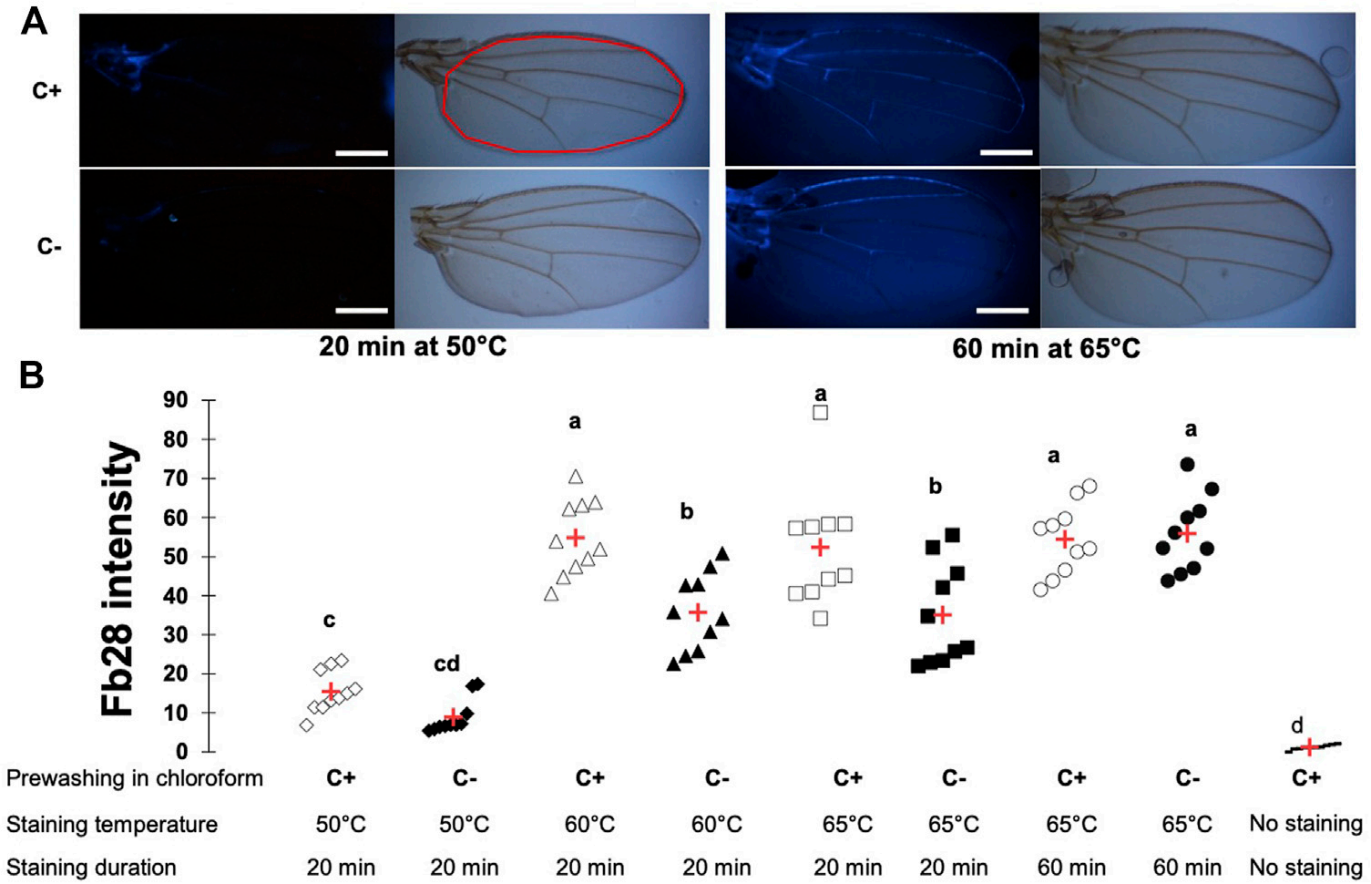
FB28 staining of the whole *Drosophila* wing requires high temperature and duration to be penetration-independent. **(A)** Pictures from wings of 48 h-old control female flies (*w1118*) stained with FB28 at 50° or 65°C during 20 min or 60 min. Upper pictures were taken in bright field. Lower pictures were taken with DAPI filter. To remove epicuticular hydrocarbons, flies were prewashed with chloroform (C+) or washed with PBS as a control (C-). Red line in the white light picture (on the right) indicates the zone used for quantification. Scale bars = 0.3 mm **(B)** FB28 intensity quantification in the whole wing excluding longitudinal veins. Data are represented as scattergrams showing all data points with the mean (red crosses). C+ and C- individuals are represented with empty and black filled markers respectively. Different marker shapes indicate different temperatures. Different letters indicate statistically significant differences (one-way Kruskall-Wallis test followed by a Conover-Iman procedure, *p*-value < 0.001). *n* = 10 in each group.

We first used the *Drosophila* wing, the simplest chitin containing tissue, as a model to develop this protocol. Importantly, without FB28 staining, *Drosophila* wings did not show any autofluorescence with our acquisition parameter (Supplementary Figure S1A). We used different staining temperature during 20 min to maximize FB28 fluorescence (435 nm). As a first concern, FB28 fluorescence intensity can result from both chitin density in the observed organ and FB28 penetration across epicuticular lipidic barrier. To discriminate between these two possibilities, we systematically prewashed some flies (C+) with chloroform to remove cuticular hydrocarbons (CHCs) and compared them with PBS-washed flies (C-) (Figure 1A). At 50°C, 60°C, and 65°C, we observed that C+ control flies (*w* ^*1118*^) were significantly more fluorescent than their PBS counterparts (Figure 1B) showing that at 65°C, the epicuticular barrier still limits FB28 penetration. Interestingly, we did not observe significant differences in C+ flies between 60 and 65°C or in C- flies between 60 and 65°C, which suggested that raising even more the temperature would not increase FB28 intensity. Moreover, higher temperature may eventually injure the tissues lowering the interpretability of the FB28 intensity. Thus, we decided to increase staining time from 20 to 60 min to possibly enhance FB28 penetration. In such conditions, we observed that FB28 stained homogeneously all inter-venous area of the wing and could not observe differences between C+ and C- flies. Of note, staining of the wing veins was highly variable, independently of staining temperature or duration, except for the anterior longitudinal vein and the thorax-proximal vein network, which were robustly stained above 50°C. Taken together, these results show that staining whole flies with FB28 during 60 min at 65°C results in a homogenous and CHCs independent FB28 intensity in the wing. Hence, we used these staining parameters in all subsequent experiments.

### Down-Regulation of Chitin-Metabolism Related Genes Affects FB28 Intensity in the Wing

**FIGURE 2:**
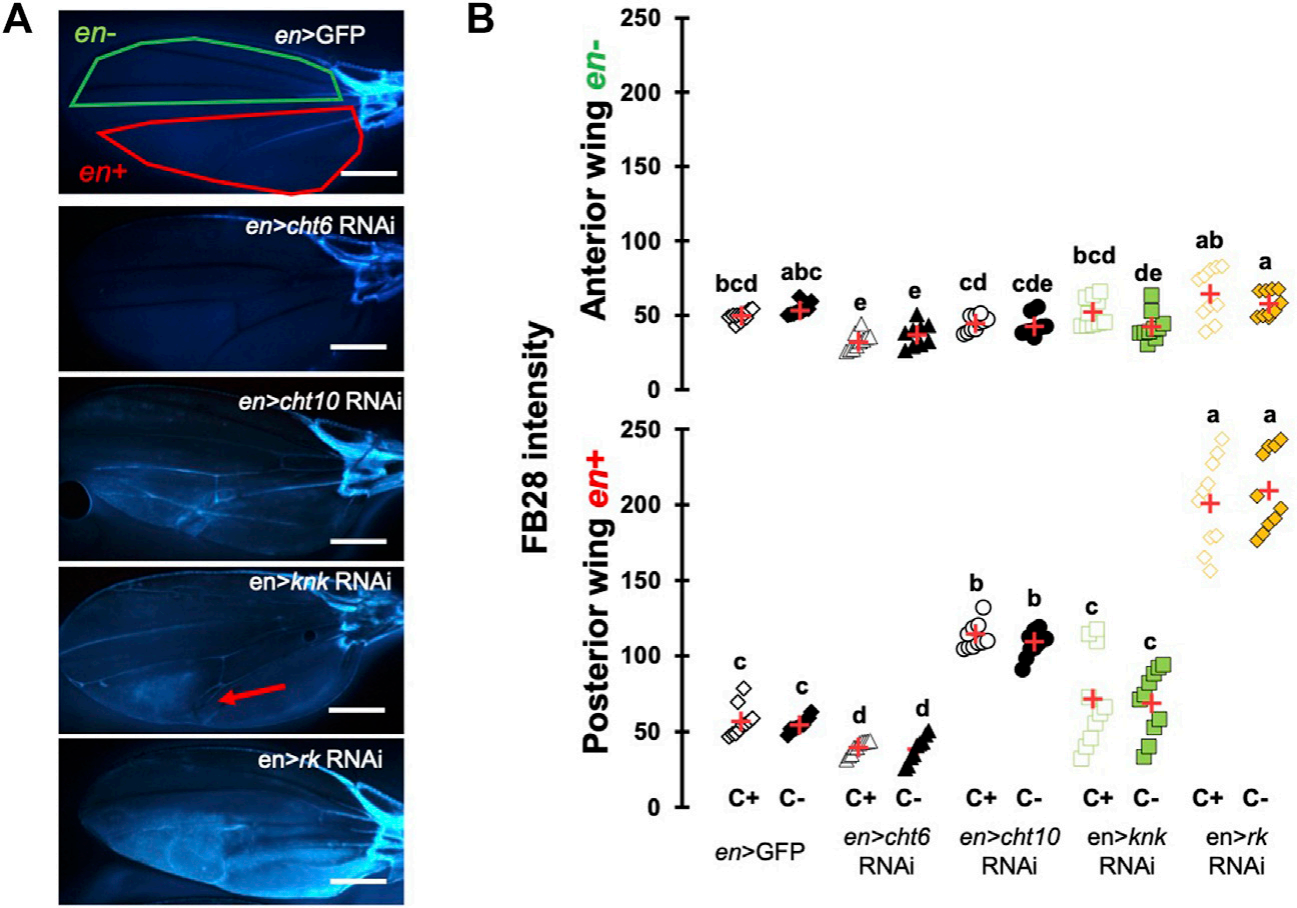
FB28 hot staining combined with transgene differentially expressed in the wing reveal how specific genes affect chitinous content. **(A)** 48 h-old female wings stained with FB28 (65°C during 60 min). Pictures were taken from flies prewashed with chloroform (C+) before staining. *en*-GAL4 was used to specifically drive RNAi expression in the posterior half of the wing. Three chitin-related genes, *chitinase 6* (*cht6*), *chitinase 10* (*cht10*) and *knickkopf* (*knk*) were down-regulated. Additionally, the gene coding for the “tanning-hormone” receptor *DLGR2* (*rk*) was down-regulated. Red line indicates the zone used for quantification of *engrailed* expressing zone (*en+*). Green line indicates the zone used for quantification of *engrailed* non-expressing zone (*en-*). Red arrow in *en* > *knk* RNAi wing indicates a vein doubling in the *en* + area. Scale bar = 0.3 mm. **(B)** FB28 intensity quantification in *en-*zone (upper panel) and *en +* zone (lower panel). Data are represented as scattergrams showing all data points with the mean (red crosses). C+ and C- individuals are represented with empty and black filled markers respectively. Different marker shapes/colors represent the different genotypes. Different letters indicate statistically significant differences (one way Kruskall-Wallis test followed by a Conover-Iman procedure, *p*-value < 0.001). *n* = 10 in each group.

To ensure that our protocol could be used to reliably quantify chitin content in a specific organ, we sought to down-regulate genes participating in chitin production or organization and hypothesized their down-regulation should affect FB28 intensity. We therefore selected the genes coding for the chitin synthase 1 (*krotzkopf verkehrt*, *kkv*), the chitin organizing protein *knickkopf* (*knk*)*,* and two chitinases, *cht10* and *cht6*, which belong to the same expression group as *kkv* in the developing wing (Sobala and Adler, 2016). Additionally, we selected the gene coding for the tanning-hormone Bursicon receptor DLGR2 (*rk*) whose down-regulation in the wing leads to cuticle misfolding independently of tanning (Flaven-Pouchon et al., 2020). To down-regulate these genes in the wing, we expressed UAS-RNAi transgenes using the *engrailed*-GAL4 (*en*-GAL4) driver. As some of these genes are lethal during early development, we always restricted RNAi expression to metamorphosis using *tub*-GAL80^TS^ transgene (see Material and Method). Conveniently, *engrailed* expression in the wing is restricted to the posterior half of the wings (Brower, 1986) allowing a direct comparison between anterior and posterior wing halves in the same fly (illustrated in Figure 2A). We first down-regulated *cht6* and *cht10* in the wing. *en* > *cht6* RNAi females showed decreased FB28 intensity in the posterior half of the wing (*en*+) compared to *en* > GFP control flies (Figure 2B). By contrast, *en* > *cht10* females showed a two-fold increase compared to *en* > GFP females thereby confirming our previous observations (Dong et al., 2020). Interestingly, anterior halves of the wings (*en*-) showed similar FB28 intensities for all genotypes except for *en* > *cht6* RNAi females. Indeed, FB28 intensity of *en-*area in these flies was significantly lower than in control flies suggesting that *cht6* may function in a non-cell autonomous manner. We then down-regulated *kkv* and *knk*; 100% of *en* > *kkv* RNAi flies lacked their posterior wing halves and could therefore not been analyzed. In *en* > *knk* RNAi females, FB28 intensity was highly variable and not significantly different from *en* > GFP females. However, we could see abnormal vein doubling in some flies confirming an organizational role of *knk* in the wing (Li et al., 2017). At last, we down-regulated the Bursicon receptor and observed a 4-fold increase in FB28 intensity compared to *en* > GFP females suggesting that Bursicon signaling strongly affects chitin metabolism. Nonetheless, Bursicon signaling is known to affect wing epidermis mesenchymal transition during wing expansion. Thus, high FB28 intensity in these flies could be due to accumulation of FB28 between dorsal and ventral cuticle layers rather than faithfully reflecting the chitin content. Taken together, these results show that down-regulation of genes participating in chitin metabolism affect FB28 intensity in the wing and establish our method as a relevant chitin quantification method for the *Drosophila* wing.

### Down-Regulation of Chitin-Metabolism Related Genes Affects FB28 Intensity in the Abdomen

**FIGURE 3:**
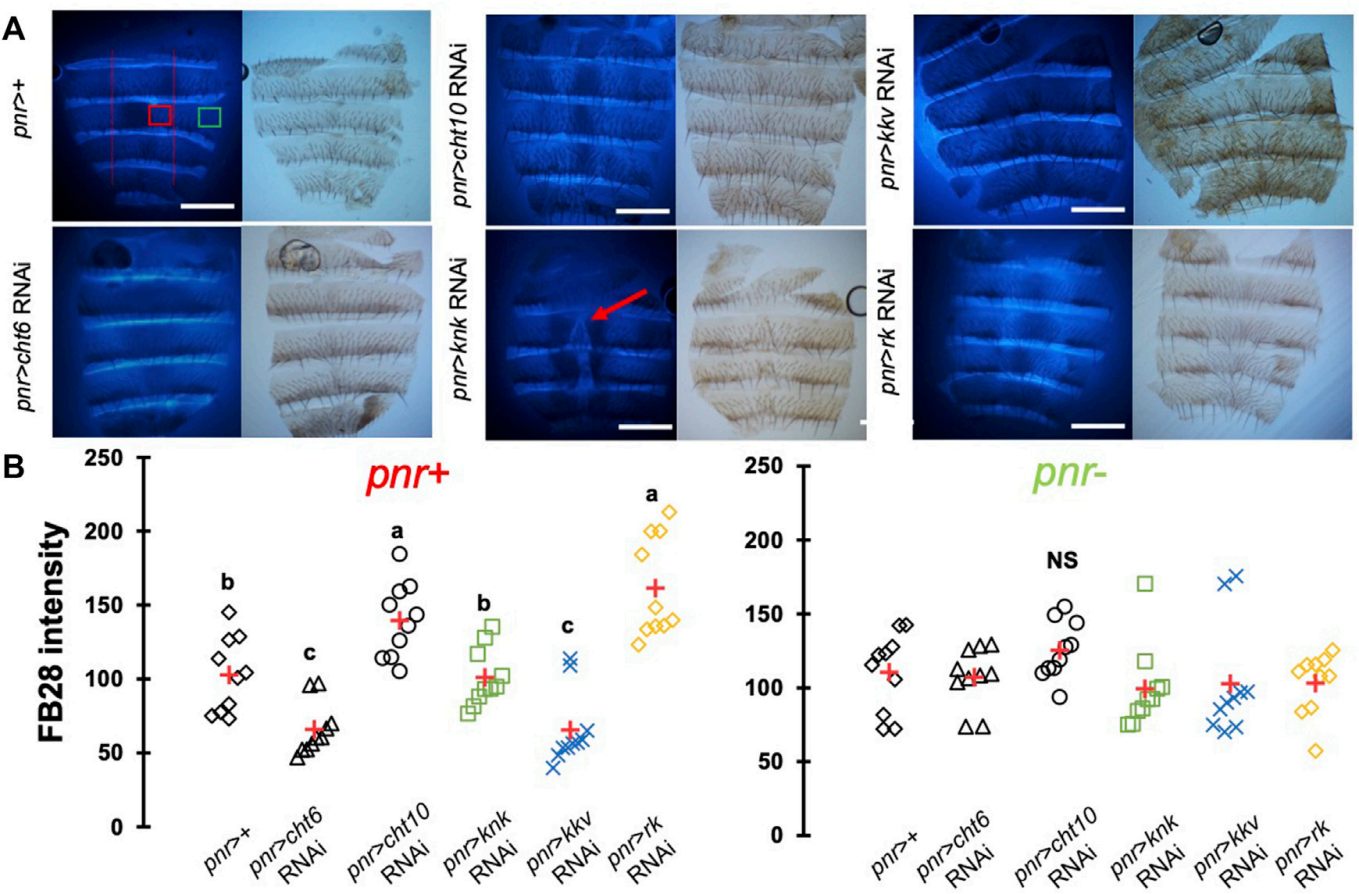
FB28 hot staining combined with transgene differentially expressed in the abdominal epidermis reveal how specific genes affect chitinous content. **(A)** 3 h-old female dorsal abdomens stained with FB28 (65°C during 60 min). *pannier* -GAL4 was used to specifically drive RNAi expression in the central part of the abdomen. Four chitin-related genes, *chitinase 6* (*cht6*), *chitinase 10* (*cht10*), *knickkopf* (*knk*) and *chitin synthase 1* (*kkv*) were down-regulated. Additionally, the gene coding for the “tanning-hormone” receptor *DLGR2* (*rk*) was down-regulated. Red line indicates the zone used for quantification of *pannier* expressing zone (*pnr+*). Green line indicates the zone used for quantification of *pnr* non-expressing zone (*pnr-*). Scale bar = 0.3 mm. **(B)** FB28 intensity quantification. FB28 intensity in *pnr +* zone (right) and *pnr-*zone (left) are shown. Data are represented as scattergrams showing all data points with the mean (red crosses). Different marker shapes/colors represent the different genotypes. Different letters indicate statistically significant differences (one way ANOVA test followed by a Tukey HSD procedure, *p*-value < 0,0001). *n* = 10 in each group.

**FIGURE 4:**
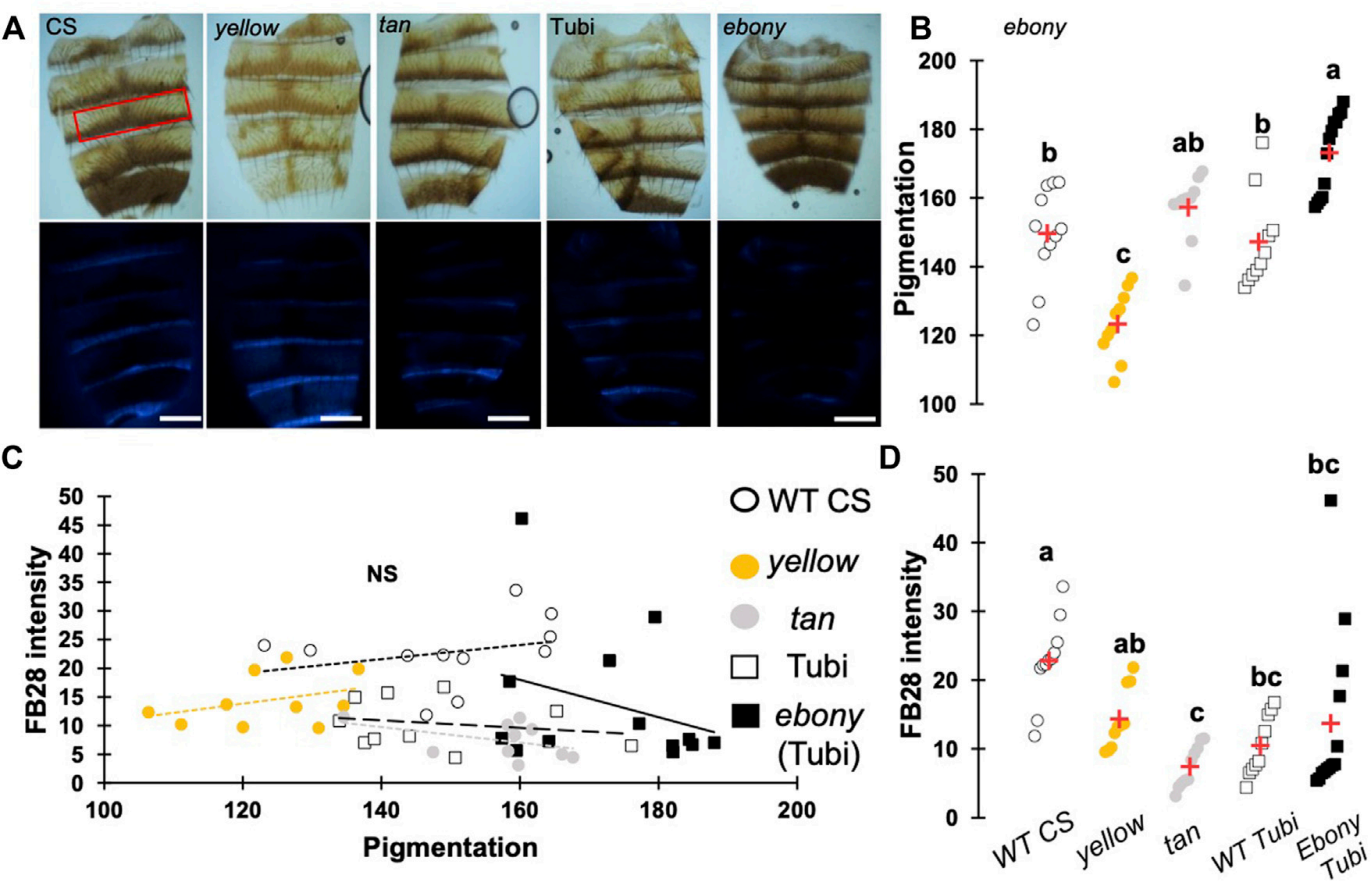
Pigmentation in 48 h-old abdomens poorly affects FB28 intensity compared to sclerotization levels. **(A)** Images of control flies (WT CS), *yellow* mutant flies and *tan* mutant flies. *ebony* mutants were obtained from wild-type Tübingen 2018 (Tubi) flies. Bright Field images (upper panel) and blue filtered images (lower panel) are shown. Scale bars = 0.3 mm **(B)** Quantification of cuticle pigmentation. Pigmentation was measured using the mean grey value in the third tergite as represented by a red rectangle in A. **(C)** Quantification of FB28 intensity expressed as a function of pigmentation. No correlation between pigmentation and FB28 were observed (Pearson correlations: −0.4 < r < 0.3, *p*-values ≥ 0.2). **(D)** Quantification of FB28 intensity alone. *tan* mutant flies showed a significantly lower FB28 intensity compared to wild-type flies. *ebony* mutant flies showed a significant, albeit variable, higher FB28 intensity. Data are represented as scattergrams showing all data points with the mean (red crosses). Different marker shapes/colors represent the different genotypes. Different letters indicate statistically significant differences (one way Kruskall-Wallis test followed by a Conover-Iman procedure, *p*-value < 0,0001). n > 10 in each group.

One major advantage of *D. melanogaster* wings to observe chitin staining is the lack of pigmentation of the non-venous cuticle. We examined whether our protocol could allow chitin quantification in melanized tissues and focused on the dorsal abdomen. After FB28 staining of 48 h-old flies, dorsal abdomens were dissected, and fluorescence was observed at 455 nm. Clearly, FB28 intensity is drastically reduced in 48 h-old abdomen suggesting dark pigmentation may affect quantification (Supplementary Figure S1C). To bypass this limitation, we used 2 h-old flies in which pigmentation is lighter. Of note, abdomen tanning in *D. melanogaster* is timely controlled by Bursicon hormone release, which starts around 1 h after adult eclosion under normal conditions (Peabody et al., 2009). After various locomotor behavior including abdominal and proboscis contraction, abdomens start tanning. To easily visualize how the selected genes down-regulation affects FB28 intensity in the abdomen, we drove RNAi expression using *pannier*-GAL4 (*pnr*) (Calleja et al., 2000). Conveniently, *pnr*-GAL4 expression pattern is restricted to the central part of dorsal abdomen epidermis and allows a direct comparison between central abdomen and the lateral sides of the abdomen as illustrated in Figure 3A. To limit impact of the melanization appearing in the posterior extremity of each segment, we restricted FB28 intensity measurement to the anterior part of the segment. Unfortunately, staining whole flies with FB28 at 65°C resulted in adverse unexpected effects. Indeed, after such treatment, soft tissues below the abdomen cuticle become highly sticky therefore making the cleaning of abdomen cuticle adventurous. To bypass this trouble, we simply pre-dissected the flies partially before staining. With the help of microdissection scissors, we cut the genitalia and removed the abdomen content. With our method, *pnr* > *cht6* RNAi females showed a decreased FB28 intensity in the *pnr +* area relatively to the *pnr-*area (Figure 3B)*.* By contrast, FB28 intensity in *pnr+* was higher in *pnr* > *cht10* RNAi abdomen confirming our observations in wings. Interestingly, *pnr* > *knk* RNAi females exhibited a more variable FB28 phenotype. In these flies, *pnr +* areas often contained alternances of dark and light FB28 intensities suggesting planar disorganization of chitin (Figure 3B). Overall, *pnr* > *knk* RNAi abdomens did not show significant differences from *pnr* > GFP controls. Down-regulation of chitin synthase *kkv* using pnr-GAL4 led to high mortality during pupariation but several escapers could be analyzed. As expected, *pnr* > *kkv* RNAi abdomens exhibited a lower FB28 intensity compared to *pnr* > GFP controls. Additionally, downregulation of *rk* resulted in a marked increase in FB28 intensity compared to *pnr* > GFP controls. Contrary to the wing, this result cannot be explained by an accumulation of FB28 between two different cuticle layers which strongly suggests that bursicon signaling affects chitin metabolism. Finally, we further studied how pigmentation levels could affect FB28 intensity by applying our protocol to flies mutant for *yellow*, *tan* and *ebony*, three genes whose roles in tanning pathways have been extensively investigated (Spana et al., 2020). In brief, the Yellow enzyme is critical to convert dopamine and DOPA into black melanins, whereas Ebony converts Dopamine into NBAD thereby addressing the Dopamine pool to NBAD sclerotization pathway (Wittkopp et al., 2009). *Tan* encodes a NBAD hydrolase which catalyzes the reverse reaction of Ebony (Wright, 1987; True et al., 2005). As expected, *ebony* and *yellow* mutant flies showed abnormal pigmentation compared to their respective controls, whereas *tan* mutant flies did not show any significant pigmentation phenotype (Figures 4A,B). However, we did not detect significant correlations between pigmentation and the FB28 signal in *yellow*, *tan* and *ebony* mutant flies neither in their respective background control flies (Canton S and Tübingen 2018 strains, respectively) indicating that pigmentation does not negatively affect FB28 intensity (Figure 4C). Thus, another factor is likely to explain FB28 intensity decrease in 48 h-old abdomens compared to immature imago abdomens. Interestingly, in *tan* mutant flies that, again, did not show any pigmentation differences compared to control flies, the FB28 intensity was strongly decreased (Figure 4D). This suggests that NBAD sclerotization could significantly impact FB28 binding to chitin. Taken together, these results establish hot FB28 staining as a reliable method to quantify chitin content in the young abdomen.

### Down-Regulation of Chitin-Metabolism Related Genes Affects FB28 Intensity in Drosophila Forelegs

**FIGURE 5:**
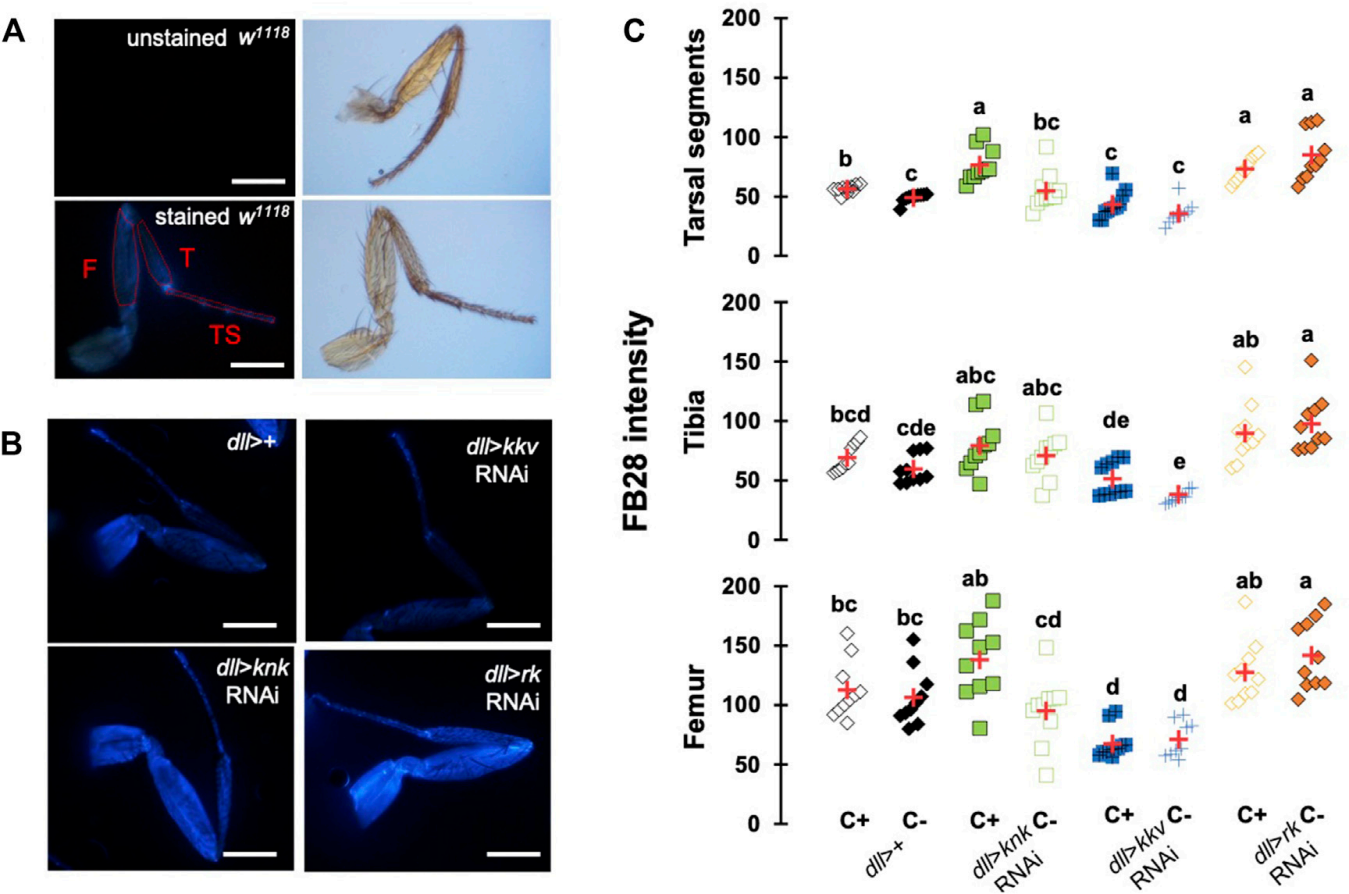
FB28 hot staining combined with transgene differentially expressed in the legs reveal how specific genes affect chitinous content. **(A)** Absence of autofluorescence in unstained *w1118* legs (upper pictures). Fluorescence is only seen after FB28 staining (lower panel). Pictures of the leg by bright field are shown on the right. Scale bars = 0.5 mm. **(B)** Female forelegs stained with FB28 (65°C during 60 min). Pictures were taken from flies prewashed with chloroform (C+) before staining. *Distalless*-GAL4 was used to specifically drive RNAi expression in the *Drosophila* leg. *dll*-GAL4 is gradually expressed from the most distal part of the legs (tarsal segments) to the junction with the thorax. Two chitin-related genes *knickkopf* (*knk*) and *chitin synthase 1* (*kkv*) were down-regulated. Additionally, the “tanning-hormone” receptor *DLGR2* (*rk*) was down-regulated. Scale bars = 0.3 mm **(C)** FB28 intensity quantification. FB28 intensity in tarsal segments (TS), tibia (T) and femur (F) were measured as illustrated by the red lines in **(A)**. Data are represented as scattergrams showing all data points with the mean (red crosses). Different marker shapes/colors represent the different genotypes. Different letters indicate statistically significant differences (one way Kruskall-Wallis test followed by a Conover-Iman procedure, *p*-value < 0,0001). *n* = 10 in each group.

To further investigate whether our method could work with other cuticular organs, we applied our method to the *Drosophila* foreleg. Legs are an interesting cuticular tissue for our method because their pigmentation is homogenous lacking strong melanization. Moreover, with hard, soft, elastic and adhesive cuticle parts, legs are among the most complex cuticular structures found in insects. Following the same rational as for wings and abdomen, we drove RNAi expression using *distalless*-GAL4, which mainly expresses in the distal tarsal segments during leg development (Gorfinkiel et al., 1997). *dll*-GAL4 expression gradually decreases in the proximal segments. As illustrated in Figure 5A, we first checked unstained legs that do not show any fluorescence. With our acquisition parameters, we did not observe fluorescence without FB28 staining. We then applied our staining method on four genotypes, *dll* > *kkv* RNAi, *dll* > *knk* RNAi, *dll* > *rk* RNAi and a *ddl*>+ control (Figure 5). We measured FB28 intensity in the three major division of the legs, namely the most distal tarsal segments, the intermediary tibia and the proximal femur (illustrated in Figure 5A). We observed in C+ *ddl*-GAL4 controls that FB28 intensity varies between these three segments (Figure 5C); femurs exhibiting the most intense staining, whereas FB28 intensity was lower in tarsal segments and the tibia (Friedman test, *p* < 0,0001, followed by a Nemenyi procedure). Interestingly, we observed a significant difference between C+ and C- *ddl*-GAL4 controls only in tarsal segments which suggests CHC quantity on the tarsal segments is higher than in more proximal parts of the leg. Generally, we observed FB28 intensity in dll > *knk* RNAi forelegs exhibited high variability. Nevertheless, FB28 intensity in C+, *ddl* > *knk* RNAi forelegs was significantly higher in tarsal segments than in C+ *dll*>+ controls. On the other hand, FB28 intensity in *ddl* > *kkv* RNAi forelegs appeared overall weaker than in *dll*>+ controls particularly in the tarsa and the femur. Finally, we observed that FB28 intensity in *ddl* > *rk* RNAi forelegs was generally stronger than in control *dll*>+ forelegs but showed a higher variability in tibia and femur segments. Taken together, these results show hot FB28 staining can be applied to the foreleg for chitin quantification and illustrate the need for a FB28 penetrability control when this method is applied.

In summary, we described in this study a rapid method to stain chitin in *Drosophila* whole body to allow chitin detection in whole tissues and provide both qualitative and quantitative information about chitin-containing structures. The down-regulation of chitin-related genes affected chitin detection in three independent tissues pointing to the robustness of our method. Particular attention must be paid to FB28 penetration versus chitin staining and we proposed a simple control to facilitate interpretation of the results.

## Discussion

### FB28 Intensity: Factors Limiting Its Interpretation

Using the wing to establish our Hot FB28 staining protocol, we determined 65°C as the optimal temperature to homogeneously stain non-venous parts of the *Drosophila* wing. Lower temperatures revealed circular staining patterns in the posterior half of the wing, whose shape is similar as in previous observations with hot Eosin Y staining (Supplementary Figure S1D, Wang et al., 2016). These observations suggest that temperatures below 65°C are not sufficient to homogeneously abolish the epicuticle lipidic barrier in non-venous part of the wings. Intriguingly, staining of the wing veins was highly variable between wings, even after 1 hour of staining or chloroform washing, except for the anterior marginal vein and the wing hinge, which was always stained even at 50°C. Interestingly, stronger staining of the wing hinge was similarly observed after Eosin Y staining suggesting that the cuticular inward barrier is particularly weak in the wing hinge (Dong et al., 2019). Moreover, high variability of distal veins staining suggests that FB28 diffusion trough the veins is limited. Higher temperatures could help homogenizing veins staining at the cost of a higher risk of tissue disruption. These differences of dye penetration within a single cuticular structure stresses the need of a penetration control. Thus, in intact flies staining, we systematically washed a separate group of flies with chloroform to remove cuticular lipids and to assess that there was no difference with water-washed flies. Overall, we did not see significant differences of FB28 between C+ flies and C- thereby confirming that staining *Drosophila* at 65°C during 60 min are appropriate conditions to rule out the possibility that FB28 penetration affects FB28 intensity. The only exception are the tarsal segments in which chloroform treatment slightly increased FB28 intensity in *en* > GFP controls. This suggests a stronger lipidic barrier is present in these distal segments which is not surprising given that *Drosophila* legs, as gustatory sensors, are daily exposed to xenobiotics (Ling et al., 2014; Gonzalez et al., 2018).

On the other hand, cuticle autofluorescence can be part of the FB28 intensity measurement. In particular, the rubber-like protein matrix resilin has excitation and emission maxima in the range of 320 and 415 nm, respectively, while FB28 shows a maximum excitation and emission at 300 and 480 nm, respectively (Vincent and Wegst, 2004). Resilin has been shown to be particularly present in insect articulations as well as wings hinges (Ardell and Andersen, 2001; Lerch et al., 2020). However, with our acquisition parameters, we did not see any fluorescence without FB28 staining (Figure 4A; Supplementary Figure S1A,B). This is likely to be explained by the low magnification we used to visualize the tissues and quantify FB28 intensity (5 × 1). Still, we recommend to always assess autofluorescence especially when visualizing the stained tissue at high magnifications.

One other concern of our method is the impact of melanization on FB28 intensity. Indeed, FB28 intensity in 48 h-old flies is strongly reduced (Supplementary Figure S1C). Thus, we focused our analyses on young dorsal abdomens (2 h after eclosion), which are less melanized. Interestingly, we observed pigmentation decrease after the down-regulation of *knk*, *rk* and in a lesser extent with *cht10* (Figure 3). However, these decreases do not correlate with FB28 intensity as the strongest pigmentation defects observes in *pnr* > *knk* RNAi did not results in a higher FB28 intensity. This suggests that low levels of pigmentation do not interfere with FB28 intensity. Conversely, FB28 staining of 48 h-old mutants of the tanning pathway did not show any correlation between pigmentation and FB28. These results suggest that FB28 intensity is hardly affected by the darkening of the mature cuticle, yet we have not applied our protocol to insects that are completely black such as *Tenebrio molitor.* Interestingly, we observed that FB28 intensity was lower in the abnormal black-melanin pattern of the wings of *ebony* mutant flies (Supplementary Figure S3). This suggests that black melanin may compete with FB28 for chitin binding or alter detection of the FB28 emission. In highly pigmented arthropods, various chemical methods have been proposed to remove melanins from the cuticle (Stuben and Linsenmair, 2009; Younes et al., 2015; Khayrova et al., 2021). However, these treatments are likely to affect the quality and/or the quantity of the chitin fibers particularly through chitin deacetylation and depolymerization (Qin et al., 2002; Younes et al., 2015). Overall, our data suggest that pigmentation is not the major concern that limits the interpretability of FB28 intensity. Importantly, the marked FB28 intensity drop that we observed between 2 h-old and 48 h-old abdomens calls for another explanation than pigmentation.

When staining a cuticular structure with FB28, we assume that there is a competition between the dye molecule which binds to chitin fibers and all endogenous chitin-binding compounds including CPRs, pigments and sclerotizing agents. Thus, the sclerotization state of a given cuticular structure could strongly affect FB28 intensity. Interestingly, we observed strong decrease of FB28 intensity in abdomens and wings of the NABD hydrolase mutant *tan* and in a lesser extent in *yellow* mutants (Figure 4D, Supplementary Figure S3). This strongly suggests that over-sclerotization of the cuticle as expected in these two mutant flies leads to a decrease in FB28 intensity. On the other hand, results we obtained by down-regulating the tanning-hormone receptor *rickets* suggest that sclerotization defects increase drastically FB28 binding. However, we recently challenged the view that Bursicon/Rk signaling in the epidermis is related to tanning (Flaven-Pouchon et al., 2020). Moreover, *rk* expression is maximal in 3 days-old pupae (Graveley et al., 2011). Thus, we cannot exclude that Bursicon/Rk signaling in the epidermis impacts chitin content through regulation of chitin-related genes during metamorphosis. Further experiments shall address the impact of *rk* down-regulation on chitin metabolism genes transcriptions. Overall, discriminating between chitin polymer availability for FB28 binding and chitin content is a real challenge in FB28 stained intact tissues. One simple solution to this concern consists in analyzing the target tissue soon after ecdysis when sclerotization is not completed which unfortunately restricts the analysis of chitin content to specific stages. Various methods have been proposed to quantify chitin after chemical extraction from biological samples (Lehmann and White, 1975; Tsurkan et al., 2021). Recently, FB28 fluorescence spectrophotometry has been proposed as a fast and efficient method to quantify chitin after mechanical extraction from insect samples (Henriques et al., 2020). We think that this method could be complementarily applied after hot FB28 staining and tissue pooling and would, because of chemical separation and release of the components, decrease the impact of sclerotization state on the chitin content quantification.

In *Drosophila*, chitin synthesis is mainly achieved by the chitin synthase *kkv*, which is highly expressed during metamorphosis (Gagou et al., 2002; Moussian et al., 2005; Sobala and Adler, 2016)*.* As expected, down-regulating the chitin synthase *kkv* during metamorphosis led to high pupal mortality with the three drivers we used (*en*, *dll* and *pnr*). However, we could obtain adult escapers of the *pnr* > *kkv* RNAi and *dll* > *kkv* RNAi genotypes, which showed a decrease in FB28 intensity. These results are particularly important as they establish that hot FB28 staining as a relevant method for chitin content detection in intact insects. Additionally, down-regulation of the chitin organizer gene *knk* overall did not change significantly FB28 intensity in wings or abdomens. Interestingly, FB28 staining was more heterogeneous in *pnr* zones (Figure 3) and venous malformation appeared in the *en* zone (Figure 2) suggesting chitin organization defects. Previous studies showed that *knk* down-regulation induced chitin fiber disorganization in the procuticle and less lamination in the wing cuticle decreasing indirectly inward barrier efficiency (Moussian et al., 2006; Li et al., 2017). With hot FB28 staining, we did not observe any significant change in FB28 intensity. FB28 intensity in *dll* > *knk* RNAi tarsal segment was slightly higher, which could be explained by inward barrier impairment rather by a higher chitin content. Of note, *knk* down-regulation decreases abdomen pigmentation as it decreases pupal wing melanization confirming that chitin organization is a determinant for pigmentation (Li et al., 2017). Together, these data are not in agreement with the finding that chitin amounts are reduced in the red flour beetle *Tribolium castaneum* and the migratory locust *Locusta migratoria* after *knk* knock-down (Chaudhari et al., 2011; Yu et al., 2021).

### Hot FB28 Staining Reveal Opposite Role of Two Chitinases

In insects, chitinases genes are more and more seen as cuticle architects and not only chitin recyclers (Pesch et al., 2016; Noh et al., 2018; Behr and Riedel, 2020). Among *Drosophila* chitinases, we recently showed that *cht10* down-regulation in the wings resulted in increase of chitin amounts possibly through uncoupling with the chitin synthase Kkv activity (Dong et al., 2020). Our results confirm these observations and suggest that the *cht10* function is conserved in various cuticular structures. Conversely, *cht6* down-regulation led to FB28 intensity decrease in all observed organs suggesting opposite roles between *cht10* and *cht6.* Interestingly, *cht6* down-regulation in the posterior wing half additionally affected the anterior wing half suggesting that *cht6* function in the wing is not tissue-autonomous, whereas *cht10* function is. These results call for a precise dissection of the localization of chitinase action, which could easily be achieved using the CRISPR tools panel available in *Drosophila* and other insect models (Rylee et al., 2018; Port et al., 2020).

In summary, our results illustrate a simple and rapid staining method of an intact insect allowing chitin detection at the tissue level. Once several aspects of cuticle biology are kept in mind, this method could be used in various insects to study the impact of genes down-regulation or insecticide applications on chitin content in whole insects.

## Data Availability

The original contributions presented in the study are included in the article/Supplementary Material, further inquiries can be directed to the corresponding author.

## Supplementary Material

Supplementary Figure S1
**(A)** No fluorescence was observed in wings in the absence of FB28 staining. *w* ^*1118*^ 48h-old female wing. Picture taken by bright field on the left. **(B)** No fluorescence was observed in abdomen in the absence of FB28 staining. *w* ^*1118*^ 2h-old female wing. Picture taken by bright field on the left. **(C)** Abdomen of 48h-old *pnr* > *rk* RNAi 48h-old females after hot FB28 staining. FB28 fluorescence is strongly reduced in the melanized center (red arrows). **(D)** *w* ^*1118*^ 48h-old female wing after hot FB28 staining at 60°C. Fluorescence patterns in posterior wings were observed in most flies and were similar to previous observations with Eosin Y penetration. Scale bars = 0.55 mm

Supplementary Figure S2 Relative FB28 intensity of the *pnr* zone in the abdomen after RNAi down-regulation of chitin-related genes. *Pnr* zone data from Figure 3 are expressed as a percentage of *pnr*—zone from the same figure. Data are represented as scattergrams showing all data points with the mean (red crosses). Different marker shapes/colors represent the different genotypes. Different letters indicate statistically significant differences (one way Krushall-Wallis test followed by a Conover-Iman procedure, *p*-value < 0.0001). *n* = 10 in each group.

Supplementary Figure S3 FB28 intensity in 48h-old wing from *yellow ebony* and *tan* mutant flies. **(A)** Bright field images (upper pannel) and blue filtered images (lower pannel) are shown. Scale bar = 0.3 mm. **(B)** Quantification of FB28 intensity in the wing. *yellow ebony* and *tan* showed a significantly lower FB28 intensity. Please note in *ebony* wings FB28 intensity follow the black melanin patterns. Data are represented as scattergrams showing all data points with the mean (red crosses). Different marker shapes/colors represent the different genotypes. Different letters indicate statistically significant differences (one way Krushall-Wallis test followed by a Conover-Iman procedure, *p*-value < 0.0001). *n* = 10 in each group.
