# Supplementary figures and images for: Fluorescent Microscopy-Based Detection of Chitin in Intact Drosophila melanogaster
Source: Front Physiol. 2022 Apr 26;13:856369. doi: 10.3389/fphys.2022.856369 (PMC9086190; doi:10.3389/fphys.2022.856369)

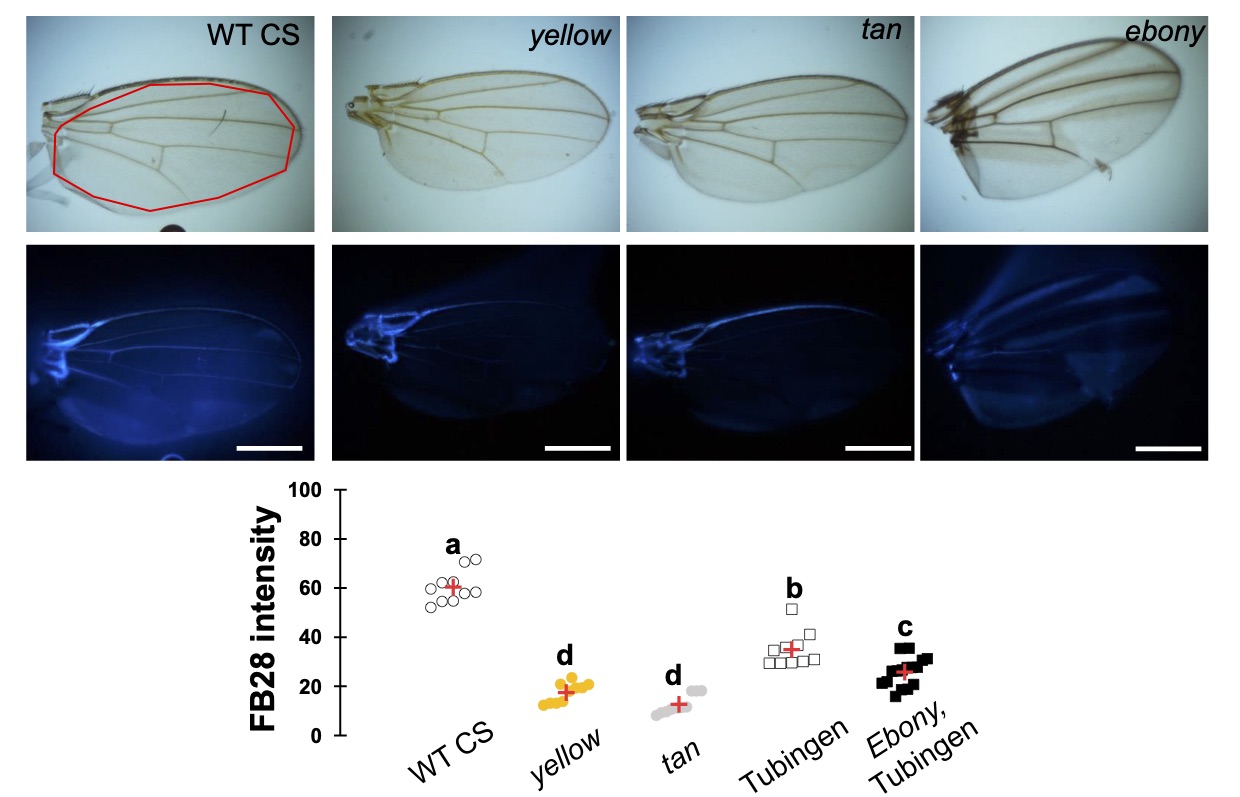

Supplement: Supplementary file 1 [file Image3.JPEG]

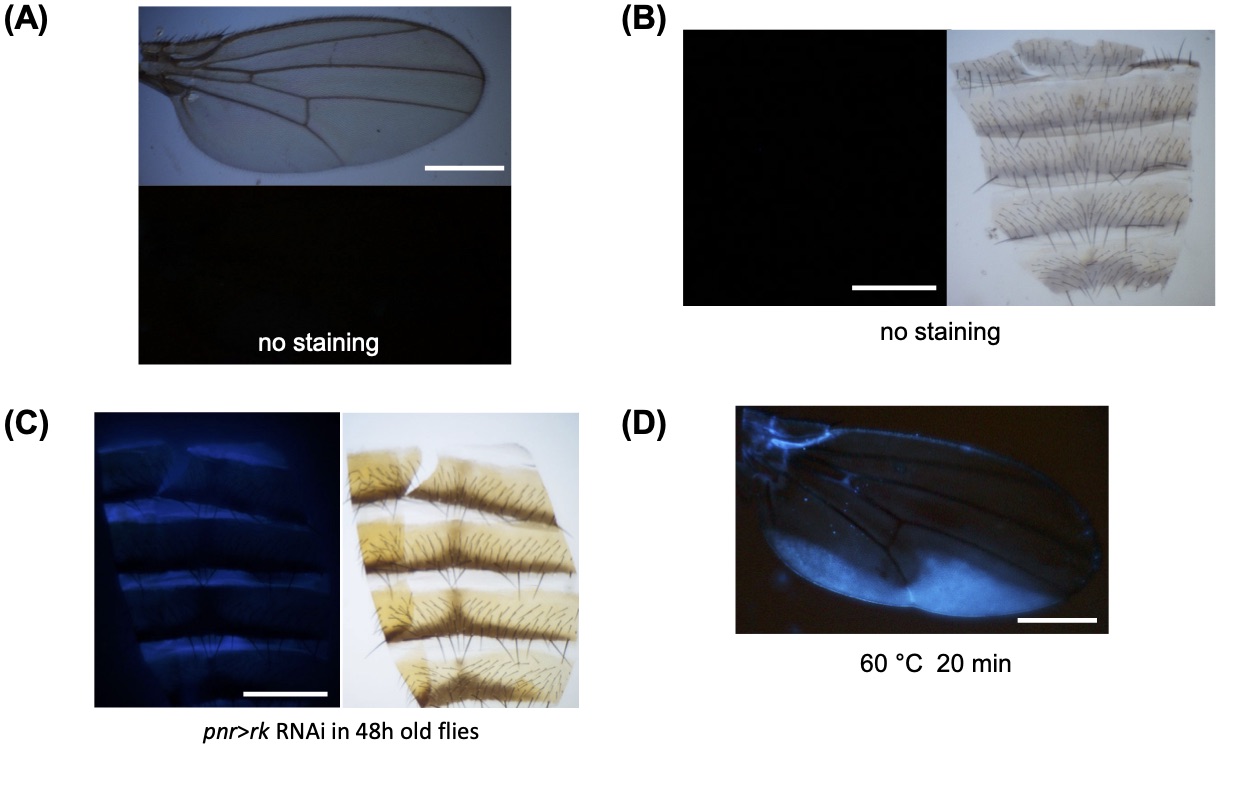

Supplement: Supplementary file 2 [file Image1.JPEG]

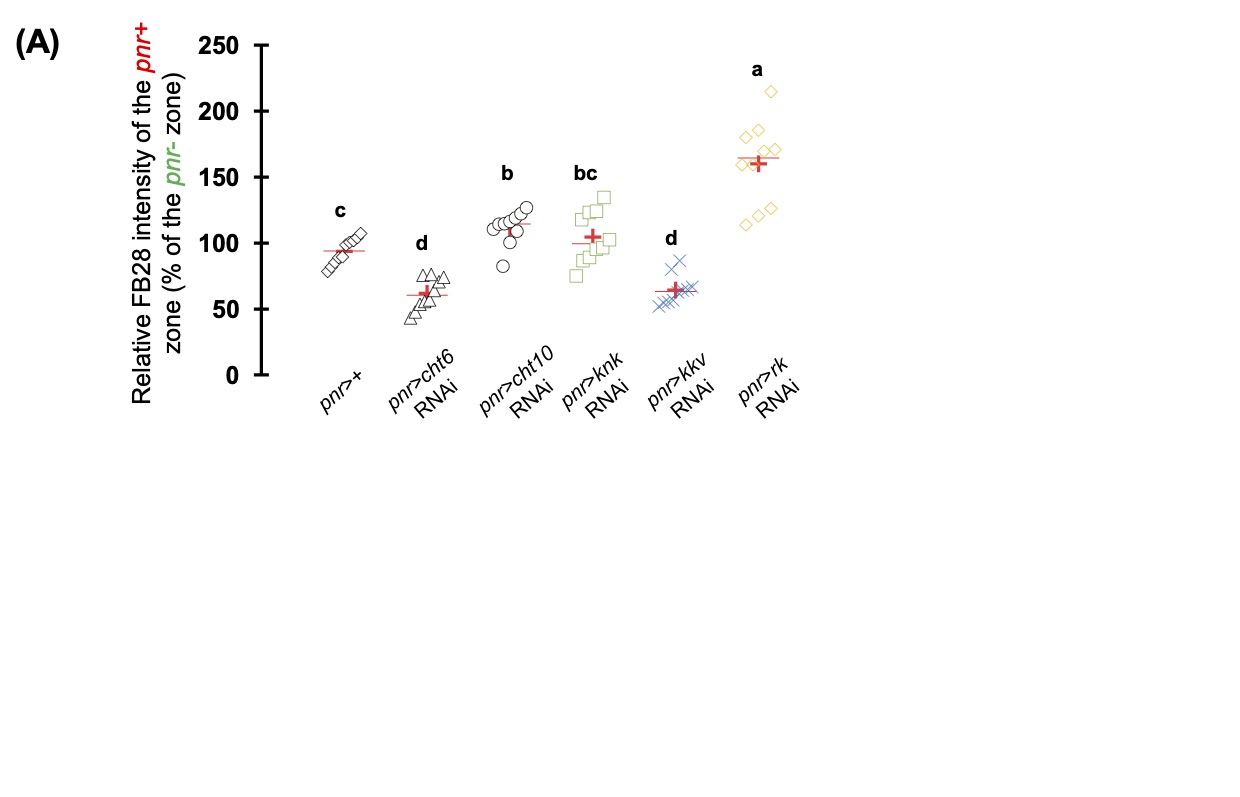

Supplement: Supplementary file 3 [file Image2.JPEG]
